# Supplementary material for: GPR88 localization to primary cilia in neurons is cell-type specific
Source: Life Sci Alliance. 2025 Dec 2;9(2):e202503366. doi: 10.26508/lsa.202503366 (PMC12672383; doi:10.26508/lsa.202503366)
Supplement: Supplementary file 1 [file LSA-2025-03366_TableS1.docx]

| **Experiment day** | **Antigen** | **Primary** | **Secondary** | **False Positives** | **Figure** |
| --- | --- | --- | --- | --- | --- |
| Day 1 | GPR88-Venus  +  AC3 | Ch anti-Venus  Rb anti-AC3 |  |  |  |
| Day 2 (morning) |  |  | Anti-ch 488  Anti-rb 594 | None |  |
| Day 2 (afternoon) | SATB2  or  DARPP-32 | Rb anti-SATB2  or  Rb anti-DARPP-32 |  |  |  |
| Day 3 | SATB2  or  DARPP-32 |  | Anti-rb 647 | Alexa 647 may detect remaining unbound anti-AC3 and anti-SATB2 or anti-AC3 and anti-DARPP-32 | **Fig 2C, Fig 2E** |
